# The relationship between telomere length and mortality risk in non-model vertebrate systems: a meta-analysis

**DOI:** 10.1098/rstb.2016.0447

**Published:** 2018-01-15

**Authors:** Rachael V. Wilbourn, Joshua P. Moatt, Hannah Froy, Craig A. Walling, Daniel H. Nussey, Jelle J. Boonekamp

**Affiliations:** 1 Institute of Evolutionary Biology, University of Edinburgh, The King's Buildings, Ashworth Laboratories, Charlotte Auerbach Road, Edinburgh EH9 3FL, UK; 2 Groningen Institute for Evolutionary Life Sciences, University of Groningen, PO Box 72, 9700 AB Groningen, The Netherlands

**Keywords:** survival, longevity, systematic review, wild, publication bias

## Abstract

Telomere length (TL) has become a biomarker of increasing interest within ecology and evolutionary biology, and has been found to predict subsequent survival in some recent avian studies but not others. Here, we undertake the first formal meta-analysis to test whether there is an overall association between TL and subsequent mortality risk in vertebrates other than humans and model laboratory rodents. We identified 27 suitable studies and obtained standardized estimates of the hazard ratio associated with TL from each. We performed a meta-analysis on these estimates and found an overall significant negative association implying that short telomeres are associated with increased mortality risk, which was robust to evident publication bias. While we found that heterogeneity in the hazard ratios was not explained by sex, follow-up period, maximum lifespan or the age group of the study animals, the TL–mortality risk association was stronger in studies using qPCR compared to terminal restriction fragment methodologies. Our results provide support for a consistent association between short telomeres and increased mortality risk in birds, but also highlight the need for more research into non-avian vertebrates and the reasons why different telomere measurement methods may yield different results.

This article is part of the theme issue ‘Understanding diversity in telomere dynamics’.

## 1. Introduction

Telomeres are highly repetitive sections of DNA that cap the ends of chromosomes in most eukaryote species, forming complexes with so-called ‘shelterin’ proteins that are essential to the maintenance of genomic integrity of linear chromosomes [1,2]. Telomeres shorten with each cell division due to the ‘end replication problem’ and in response to cellular stressors including oxidative stress, and induce cellular senescence when they shorten below a critical threshold [1,3,4]. Telomeres can be restored via several mechanisms, the most widely studied being the action of the enzyme telomerase [1,3]. Telomerase expression appears to be suppressed in adult somatic tissue in many large-bodied endothermic vertebrates, including humans [5]. Telomere attrition has been identified as one of nine ‘hallmarks of ageing’ [6] and while the role of telomere shortening in cellular senescence is beyond doubt, the evidence that it plays a causal role in senescence in otherwise healthy animals is currently weak [7]. However, there is mounting evidence in humans that average telomere length (TL), typically measured in blood cells, represents an important biomarker of health and ageing [3]. Leucocyte TL declines with age in humans [8] and meta-analyses have recently shown that in adult humans shorter average TL is associated with increased risk of type 2 diabetes, cardiovascular disease, cancer and follow-up mortality [9–12]. Although the majority of non-human research into telomere biology has been performed in laboratory rodents, studies beyond model organisms are crucial if we are to understand the evolutionary and environmental factors responsible for the diversity of TLs and levels of telomerase expression observed among species [13,14].

There is a rapidly growing literature exploring telomere dynamics and their significance for organismal function and fitness in non-human vertebrates and, in particular, in wild bird systems [15–17]. TL has been proposed as an important biomarker within evolutionary ecology and animal welfare because it may reflect an individual's cumulative experience of environmental stress and investment in growth or reproduction [17–19]. This leads to the expectation that shorter TL will predict raised subsequent mortality risk, without telomeres necessarily being causally involved in death, due to increased somatic damage associated with environmental stress and reduced investment in somatic repair [17,19]. In humans, evidence is also emerging that TL is both highly repeatable over time within individuals and highly heritable [20,21]. This raises the further possibility that individual differences in TL set at birth are maintained throughout life and are associated with consistent differences in physiological function or state and organismal lifespan. Although determining the relative importance of TL at birth and TL shortening over life for organismal health and fitness remains a major outstanding challenging within telomere biology [17,20], a crucial first step towards this goal is to establish whether an overall association between TL and mortality risk is evident in non-human species and how and why such an association might vary across species.

Several studies have reported significant associations between average blood cell TL and the risk of subsequent mortality in both wild and captive populations [22–25]. However, this emerging literature also contains numerous examples of studies that test for but do not find evidence to support a relationship between TL and mortality risk [26–28]. Thus, the generality of the relationship between TL and mortality is currently unclear outside of studies of humans and laboratory rodents. A number of factors may contribute to the variation observed in the relationship between TL and mortality in these non-model organisms. First, studies invariably apply one of two methodologies—quantitative PCR (qPCR) or terminal restriction fragment analysis (TRF)—which differ in accuracy and throughput, with the former providing the average amount of telomeric DNA within a sample on a relative and non-comparable scale and the latter providing information on the range of TLs within a sample in kilobase units [29,30]. The life history of the species in question, in particular its life expectancy under natural conditions, is also expected to play an important role in shaping the evolution of telomere dynamics [13,14,19]. Ecological studies of TL also vary considerably in the duration of the study follow-up time, from weeks [31] to over a decade [22], and typically involve investigating TL and survival in either young animals or adults rather than both. Furthermore, while sex differences in TL observed in humans and laboratory rodents have been proposed to underpin sex differences in longevity, the effect of sex on the relationships between TL and mortality has rarely been investigated [32,33]. Here, we undertake the first formal literature search and meta-analysis to test whether there is a consistent association between short TL and increased subsequent mortality risk in vertebrates other than humans and model laboratory rodents. In addition, we use meta-regression analyses to investigate potential sources of variation in this association across studies including methodology, life stage at sampling, follow-up period and sex.

## 2. Methods

### (a) Literature search

**Figure 1.**
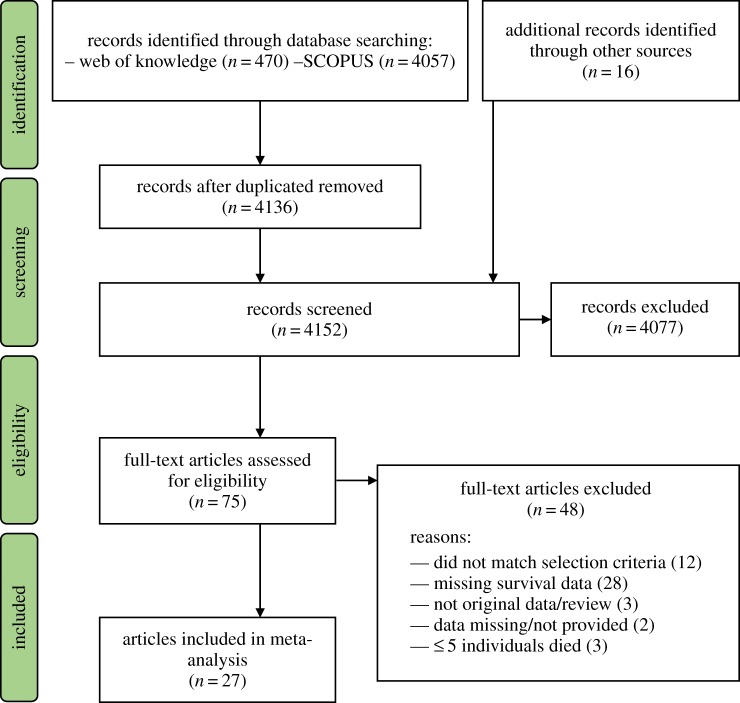
Preferred Reporting Items for Systematic Reviews and Meta-Analyses (PRISMA) flow diagram for identification and inclusion of studies in the meta-analysis. We present the number of papers identified through key word database searching in addition to records identified through other sources. Papers were excluded during initial screening phases and reasons for exclusion provided for those papers that reached the final full-text eligibility screening. (Online version in colour.)

Data for our meta-analysis were collected using ISI Web of Science and SCOPUS databases with the following search string: ‘telom* AND surviv* OR longevity/lifespan/life span/life expectancy/mortality/fitness'. Additional papers were identified in two ways: (i) backward and forward searching was carried out on citations of the first paper showing an association between TL and survival in a non-model vertebrate [23]; (ii) screening the authors' own reference list, created from Google Scholar email alerts containing the keyword ’telomere’, for relevant papers. The last database search was carried out on 6 February 2017, although Google Scholar alerts were continuously checked and papers published up until May 2017 were included. We included studies published as part of PhD theses that were available online, but otherwise excluded studies that had not yet been published in peer-reviewed journals. Overall, these searches identified 4152 papers for potential inclusion in our meta-analysis (figure 1).

Since our focus was on non-model vertebrate systems, we excluded studies involving human subjects, genetically modified or inbred laboratory strains of mice and non-vertebrate species. We excluded studies that did not report original empirical data (i.e. reviews or computer simulations) and those in which an association between TL and mortality or longevity was not reported. Our initial screening based on titles and abstracts of papers in the database led to the exclusion of 4077 papers (mostly studies of humans and model laboratory organisms), with 75 papers retained for more detailed interrogation of eligibility (figure 1; electronic supplementary material, table S1). The full text of these papers were downloaded and a further 46 excluded. This left 29 studies that were suitable for inclusion in our meta-analysis, and we were able to obtain data from 27 of these studies (see electronic supplementary material, table S1 for full details of reasons for exclusion). Although the majority of papers read in full measured TL in blood cells, several did measure TL in other tissues but none of those studies provided suitable data or analyses of lifespan or survival for inclusion our meta-analyses. Indeed, most studies read in full were excluded because suitable data on survival of individuals were lacking, but we also excluded three studies that reported an association between TL and survival but in which fewer than five individuals died (<10% of study population), as power to detect TL–mortality risk relationships would be extremely limited in such cases (see electronic supplementary material, table S1).

During our search, we noted a great deal of heterogeneity in the manner in which analyses of TL–mortality risk relationships were conducted and reported, as well as in the way TL was measured (qPCR or TRF methodologies). To maximize our ability to detect an overall association between TL and survival across studies, and to identify the factors responsible for variation in this association among studies, we decided to obtain raw data for each study and analyse the TL–mortality relationship in a standardized way. For studies in which raw data were not available online, we contacted the corresponding authors requesting either that they provided us with the raw data used in the relevant analyses, or that they performed standardized analyses using an R script that we provided (electronic supplementary material, file S1). We were able to obtain raw data or standardized measures of TL–mortality risk associations from 27 studies identified from 20 different species: 17 bird species, three reptiles and one mammal (table 1).

**Table 1.** List of studies included in the meta-analysis with samples sizes (*N*), effect sizes, expressed as the natural logarithm of the hazard ratio of TL and associated standard error (s.e.) alongside information on moderator variables tested (see §2 for details).

study	ref.	class	order	species	*N*	ln hazard ratio	s.e.	follow-up	TL method	age group	log lifespan
Angelier (2013)	[34]	Aves	Passeriformes	*Setophaga ruticilla*	36	−1.100	0.460	1	qPCR	adult	2.312535
Asghar (2015)	[35]	Aves	Passeriformes	*Acrocephalus arundinaceus*	100	−0.293	0.113	23	qPCR	juvenile	2.312535
Barrett (2013)^a^	[22]	Aves	Passeriformes	*Acrocephalus sechellensis*	203	0.064	0.071	15	qPCR	adult	2.833213
Barrett (2013)^a^	[22]	Aves	Passeriformes	*Acrocephalus sechellensis*	203	−0.414	0.192	1	qPCR	adult	2.833213
Bauch (2014)	[36]	Aves	Charadriiformes	*Sterna hirundo*	181	−0.140	0.113	4	TRF	adult	3.496508
Beaulieu (2011)	[26]	Aves	Sphenisciformes	*Pygoscelis adeliae*	72	0.036	0.313	3	qPCR	adult	2.772589
Belmaker (2016)	[37]	Aves	Passeriformes	*Tachycineta bicolor*	107	−0.054	0.124	1	TRF	adult	2.493205
Bize (2009)	[24]	Aves	Apodiformes	*Apus melba*	96	−0.348	0.126	6	qPCR	adult	3.258097
Boonekamp (2014)	[38]	Aves	Passeriformes	*Corvus monedula*	241	−0.023	0.149	8	TRF	juvenile	3.010621
Caprioli (2013)	[39]	Aves	Passeriformes	*Hirundo rustico*	60	−0.014	0.136	11	TRF	juvenile	2.772589
Fairlie (2016)^a^	[40]	Mammalia	Artiodactyla	*Ovis aries*	87	−0.262	0.315	12	qPCR	adult	3.126761
Fairlie (2016)^a^	[40]	Mammalia	Artiodactyla	*Ovis aries*	116	−0.405	0.206	1	qPCR	juvenile	3.126761
Foote (2009)	[41]	Aves	Procellariiformes	*Macronectes halli*	36	−0.060	0.249	8	TRF	adult	3.688879
Foote (2011)	[42]	Aves	Procellariiformes	*Macronectes giganteus*	47	−0.327	0.195	8	TRF	adult	3.688879
Reichert (2017)	[43]	Aves	Procellariiformes	*Diomedea exulans*	56	−0.227	0.224	12	qPCR	adult	3.912023
Geiger (2012)	[44]	Aves	Sphenisciformes	*Aptenodytes patagonicus*	36	−2.198	0.524	1	qPCR	juvenile	3.258097
Haussmann (2005)	[23]	Aves	Passeriformes	*Tachycineta bicolor*	22	−0.741	0.308	4	TRF	juvenile	2.493205
Heidinger (2012)	[25]	Aves	Passeriformes	*Taeniopygia guttata*	99	−0.420	0.108	8.7	qPCR	juvenile	2.484907
Olsson (2011)	[33]	Reptilia	Squamata	*Lacerta agilis*	126	−0.071	0.084	25	TRF	adult	2.079442
Ouyang (2016)	[45]	Aves	Passeriformes	*Tachycineta bicolor*	74	−0.034	0.127	3	TRF	adult	2.493205
Reichert (2014)	[27]	Aves	Passeriformes	*Taeniopygia guttata*	50	−0.161	0.227	1	qPCR	adult	2.484907
Reichert (2015)	[46]	Aves	Passeriformes	*Taeniopygia guttata*	65	−0.624	0.296	1	qPCR	juvenile	2.484907
Salomons (2009)	[47]	Aves	Passeriformes	*Corvus monedula*	48	−0.076	0.219	4	TRF	adult	3.010621
Stier (2014)	[48]	Aves	Sphenisciformes	*Aptenodytes patagonicus*	82	−0.352	0.190	1	qPCR	juvenile	3.258097
Sudyka (2014)	[28]	Aves	Passeriformes	*Cyanistes caeruleus*	56	−0.036	0.145	2	qPCR	adult	2.681022
Taff (2017)	[49]	Aves	Passeriformes	*Geothlypis trichas*	89	−0.296	0.167	4	qPCR	adult	2.442347
Ujvari (2009)^a^	[50]	Reptilia	Squamata	*Liasis fuscus*	50	0.477	0.182	10	TRF	juvenile	3.288402
Ujvari (2009)^a^	[50]	Reptilia	Squamata	*Liasis fuscus*	20	0.117	0.278	3	TRF	adult	3.288402
Ujvari (2016)	[51]	Reptilia	Squamata	*Chlamydosaurus kingii*	72	−0.496	0.254	2	qPCR	adult	2.292535
Watson (2015)	[31]	Aves	Procellariiformes	*Hydrobates pelagicus*	59	−1.260	0.430	0.2	qPCR	juvenile	3.520461

^a^Multiple estimates associated with different age groups or follow-up times in our analysis.

### (b) Effect size extraction

For each of the 27 included studies, the following data were available: individual identity, age at blood sampling, sex, TL at sampling, sampling date, final date where survival was determined and survival status (survived = 0, dead = 1). Within each study, TL was mean centred and standardized to unit standard deviation prior to inclusion in analyses to create similarly scaled TL distributions among studies. We then applied Cox proportional hazard regression analysis in R using the package *survival* [52] including TL as an explanatory variable. We defined start time as the time of TL sampling and end time as the follow-up time at which survival was determined. This information was used to determine the hazard ratio of TL relative to baseline mortality. Final effect sizes were expressed as the natural logarithm of the hazard ratio for mortality (ln HR). As ln HR provides a measure of risk of death, a negative effect indicates that individuals with long TL on average are less likely to die in comparison to individuals with short TL. Hazard ratio estimates and associated standard errors were extracted, either by ourselves or the authors (using the R script in electronic supplementary material, file S1).

### (c) Meta-analysis

We conducted our meta-analysis using the *metafor* package [53] in R, to investigate the relationship between TL and survival. We used a random-effects design fitted with restricted maximum log likelihood and used 1/s.e.^2^ as weighting factor [54], where s.e. was the standard error associated with the ln hazard ratio from the Cox regression model. We tested for evidence of publication bias using Kendall's tau test-statistics and through visual inspection of funnel plots. We used *Q*-tests to evaluate study heterogeneity.

We subsequently investigated potential sources of heterogeneity by including moderator variables in the meta-analysis. We extracted the following moderator variables for each study: TL measurement method (TRF or qPCR), the age group of the study animals (categorized as ‘young’ if ≤1 year and ‘adult’ if >1 year), the length of the follow-up period in years after TL measurement, and the log transformation of each species' maximum recorded lifespan (from the AnAge database: http://genomics.senescence.info/species/). To crudely test for a phylogenetic signal, we tested class, order and species separately as moderators (20 species, six orders, three classes; table 1). Two of the studies reported separate associations between TL and survival in both age classes [40,50], while one reported associations based on two different follow-up periods [22]. We generated and included two estimates for each of these studies, resulting in a total of 30 hazard ratio estimates in the meta-analysis (table 1). Although we categorized studies based on wild or captive animals, only three were based on captive populations and so we did not investigate this moderator further [25,27,46]. Although not all papers specifically reported on sex differences in the association between TL and survival, 25 out of 27 provided complete data on the sex of individuals. Of these, three studies included only females [23,31,40], two included only males [34,49] and the remaining 20 included both sexes. To assess the effect of sex on the association between TL and survival, we re-ran Cox regression models for these latter 20 studies separately for each sex. This generated a total of 46 sex-specific hazard ratio estimates, allowing us to test sex as a moderator variable. Individual moderator effects were evaluated using either *Q*-tests for effects of class, order or species and *z*-tests for all other moderator variables.

## 3. Results

**Figure 2.**
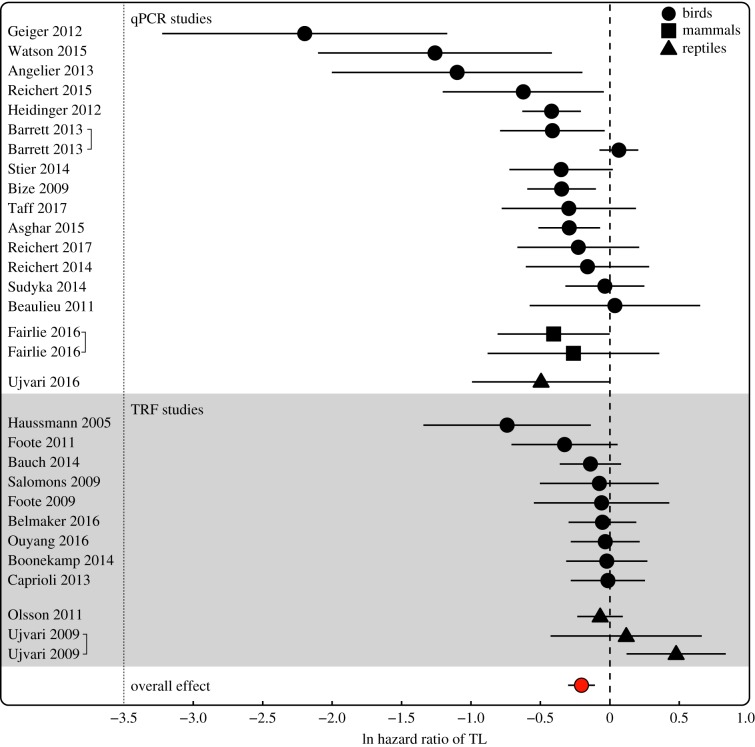
Forest plot of effect sizes (natural logarithm of the hazard ratio for standardized telomere length) and associated 95% confidence intervals. The overall effect size is shown in red, with estimates grouped by measurement method and with vertebrate class indicated by symbol shape (circle: birds, square: mammals, triangle: reptiles). (Online version in colour.)

**Figure 3.**
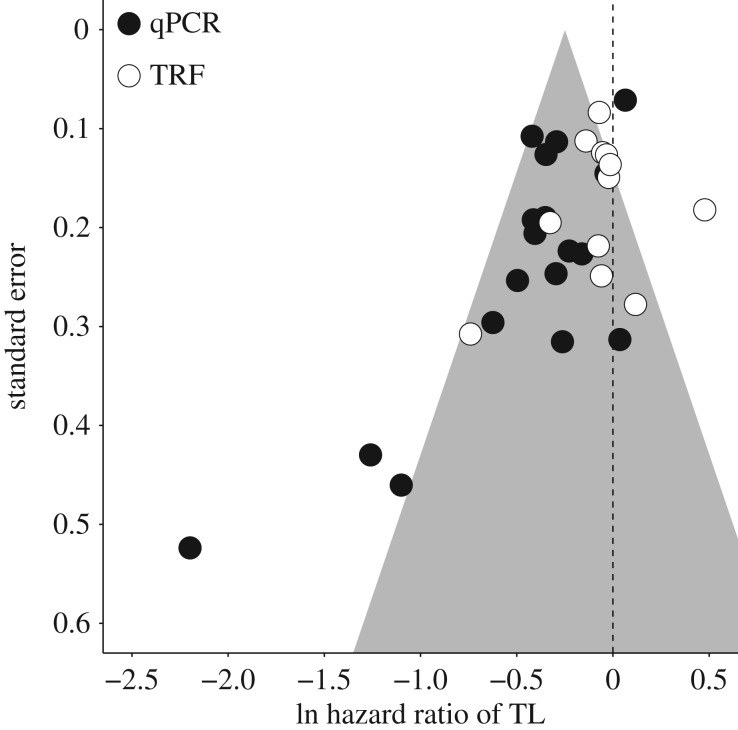
Funnel plot relating the study standard error to effect size. Open circles denote qPCR-based studies, filled circles denote TRF-based studies.

Overall, the hazard ratio associated with TL was significantly negative, supporting a decreased mortality risk with increasing TL across studies (mean ln HR = −0.205 ± 0.049 s.e., *p* < 0.001, figure 2). However, there was evidence for publication bias (Kendall's tau = −0.310; *p* = 0.016; figure 3). Visual inspection of a funnel plot relating effect size to s.e. (figure 3) revealed that this bias was primarily driven by three qPCR-based studies with small sample sizes with strongly negative hazard ratios (ln HR > −1: [31,34,44]). To establish whether this bias influenced the overall association between TL and mortality risk, we re-ran the models without these three studies; the overall association remained significant (−0.162 ± 0.044; *p* < 0.001) and the Kendall's tau statistic became non-significant (−0.134; *p* = 0.341). We also applied the ‘trim and fill’ method [55] to examine the sensitivity of the results to publication bias and found that the overall association became substantially weaker and remained marginally significant (−0.108 ± 0.062 s.e.; *p* = 0.083).

**Figure 4.**
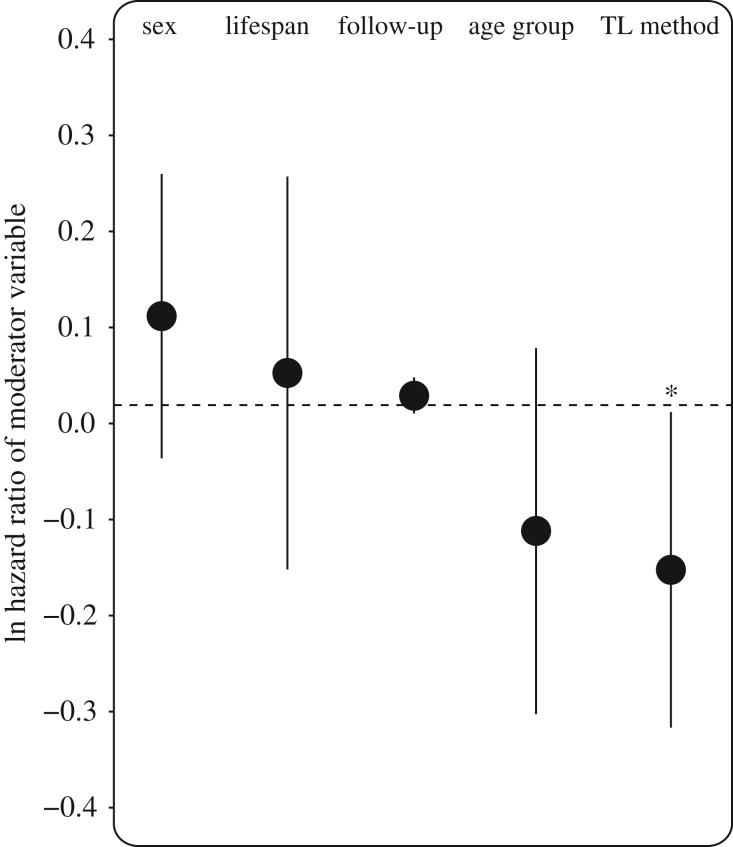
Natural logarithm of hazard ratio of moderator variables. Grouping differences are expressed as follows: Sex: male–female; TL method: TRF–qPCR; age group: adult–juvenile. Follow-up and the log of lifespan were tested as continuous variable in years. Bars indicate the 95% confidence intervals.

There was significant heterogeneity among study effect sizes (*Q*_(d.f.__=__29)_ = 77.77; *p* < 0.001) indicating substantial variation in TL–mortality risk associations among studies. We investigated the extent to which phylogeny, study follow-up period, sex, TL measurement method and age group at sampling reduced the observed study heterogeneity. We tested species, order and class as phylogenetic moderators in separate models and, although none was significant overall (QM_(d.f.__=__19)_ = 20.88; *p* = 0.405 and QM_(5)_ = 6.035; *p* = 0.419 and QM_(2)_ = 3.89, *p* = 0.143, respectively), *post hoc* comparisons within the class model suggested that the strength of the association was marginally weaker in reptiles than birds (difference bird–reptile: 0.255 ± 0.139 s.e., *p* = 0.066). The fact that there were only three reptile studies in our meta-analyses meant there was limited power to dissect this trend further, but visual inspection of figure 2 suggests it could be driven by a positive TL–mortality risk association from a TRF-based study of water pythons (*Liasis fuscus*) [50]. We did not detect a significant difference between the sexes (0.093 ± 0.076; *p* = 0.22) and there was no significant relationship with maximum lifespan (0.034 ± 0.104; *p* = 0.75), follow-up period (0.010 ± 0.006; *p* = 0.118) or age at sampling (0.131 ± 0.097; *p* = 0.177; figure 4). However, telomere measurement method explained a significant portion of the observed study heterogeneity (11.2%). The negative association between TL and mortality risk was significantly stronger in studies using qPCR relative to TRF methods (difference TRF–qPCR: −0.260 ± 0.090; *p* = 0.004; figure 4).

To explore this further, we split the data by method and ran separate models without moderators. Within the qPCR studies, the TL–mortality risk association was highly significantly negative with significant heterogeneity among studies (−0.331 ± 0.068, *p* < 0.001; *Q* = 52.41, *p* < 0.001), while there was no significant overall association or evidence for heterogeneity in TRF studies (−0.056 ± 0.042, *p* = 0.183, *Q* = 16.62, *p* = 0.120). Since we detected a non-significant trend for a weaker TL–mortality risk association in reptiles, but have very limited power to differentiate effect sizes in non-avian classes (table 1), we re-ran our analyses including only bird studies. We found a similarly negative and significant overall effect (−0.224 ± 0.050; *p* < 0.001), but the method moderator effect became weaker and marginally non-significant in this dataset (difference TRF–qPCR: −0.184 ± 0.099; *p* = 0.063), suggesting that the strongly positive TL–mortality risk estimate from the python study was at least in part responsible for the method effect we observed.

## 4. Discussion

We found that short TL was associated with increased risk of mortality, and that this result is robust to correction for evident publication bias. While many recent papers have cited a handful of salient examples as evidence for such a general pattern, here we provide the first formal test to support a TL–mortality association across studies of non-human vertebrates. While our results provide important overarching support for the importance of TL as a biomarker within ecological and evolutionary studies, they also highlight several important issues for the rapidly emerging literature on telomere dynamics in non-model vertebrate systems. First, the overall effect size was small and showed considerable heterogeneity among studies. Evidence of publication bias in our analyses argues that particular effort should be directed at supporting the unbiased publication of both non-confirmative and confirmative findings in future research in this area. Second, the lack of suitable studies in mammals and ectothermic vertebrates means that we cannot currently generalize the overall TL–mortality risk association beyond birds, and more research effort into the links between TL and fitness is clearly required in non-avian vertebrate species. Finally, the presence of an unexpected effect of telomere measurement method on the strength of the TL–mortality risk association highlights the recurrent issue within this field posed by the application of differing methodologies across studies. More studies which apply both qPCR and TRF methods side-by-side within single studies are required to help understand the reasons for these method effects.

Our meta-analysis provides strong support for the proposition that short TL predicts increased mortality risk in birds, but the generality of this pattern across all vertebrate species remains an important and open question. Although telomeres perform a crucial conserved function across eukaryotes, telomere dynamics and levels of expression of telomerase in somatic tissues vary widely among taxa [5]. In ectothermic vertebrates, telomerase expression is frequently observed in somatic tissues and this is thought to be due to the indeterminate growth of many of these taxa [5]. There is evidence for complex telomere dynamics with age in ectotherms, with studies of wild reptiles demonstrating increases in average blood cell TL through early life followed by a plateau or decline [50,51]. Recent studies of laboratory fish suggest that somatic telomerase expression can be detected throughout life, and that TL and telomerase expression can increase during development and adolescence before plateauing or declining in later adulthood [56,57]. In mammals, variation in telomerase expression has been attributed to body size and cancer prevention: telomerase is repressed in somatic cells in larger-bodied species but not in smaller ones [13,14]. In birds, although variation in somatic telomerase expression has been observed [58], it seems to be widely accepted that somatic telomerase expression is limited and the situation resembles that in large-bodied mammals [5]. These differences are further complicated by the fact that mammals have enucleated red bloods cells and so blood cell TL is measured in leucocytes, while in other vertebrate classes it is measured very predominantly in erythrocytes. In our analyses, we found a suggestive trend for weaker TL–mortality risk associations in reptiles compared to birds that may have been driven by a single outlying study. That study, of water pythons, found that in adults long, rather than short, TL was significantly associated with increased mortality risk [50], suggesting that an inversion of the relationship we observed more widely in birds may occur in some ectothermic species. It is worth noting that the observed negative relationship in pythons could be driven by age differences in TL among recaptured and non-recaptured adults, if younger adults have both longer telomeres and are less likely to survive to recapture than older adults. However, a very recent study—published after our literature search was completed—showed a similar effect in wild Atlantic salmon: juveniles with short TL were more likely to survive to recapture at return migration to natal rivers in adults [59]. Studies from a wider range of mammalian and ectothermic vertebrate species relating TL to fitness will help understand when and why such positive associations might occur, and it may prove that null or positive association is the norm in small mammals and ectotherms, in which somatic telomerase expression may counteract any signal of cumulative stress or past life history on TL shortening.

We found that studies using qPCR methods detected a stronger overall association between TL and mortality risk, and greater heterogeneity in this relationship compared to TRF studies. This method difference in the overall association is surprising given that the qPCR method has been demonstrated to be less technically repeatable than TRF [60]. However, we note that technical variation in telomere assays is likely to vary greatly across laboratories and is rarely reported in a consistent enough way to make accounting for it possible in meta-analysis. As in the human literature, the qPCR methodology is progressively becoming the dominant method in non-model vertebrate studies, presumably because it is higher throughput and less expensive [29,30]. One possible driver of the stronger overall effect in qPCR studies could be differential publication bias among studies using this method, which is suggested by the presence of three qPCR-based studies outside the lower left end of the funnel plot (figure 3). The risk of publication bias could be expected to be stronger within qPCR studies, which are generally easier to set up and quicker and cheaper to run, compared to TRF studies [29,30]. TRF data are not just harder won in the laboratory, but also more informative as they measure the variation in TL within a sample, allowing a wider range of questions to be addressed [29,30]. Thus, researchers using TRF may be more inclined and readily able to publish non-confirmatory and opposing findings. However, it is also important to keep in mind that the two methods measure slightly different things: TRF quantifies the mean length of telomere sequence in the sample, qPCR the total quantity of telomere sequence present. It is possible that, because TL distributions within samples may be highly skewed and this will affect TRF measures more than qPCR measures, the latter method may provide estimates of TL that are better predictors of organismal health and fitness. Finally, we found hints that the method effect could be driven by taxonomic bias in our estimates. We found suggestive evidence that the effect was in part driven by a TRF-based reptile study that documented a significant positive association between TL and mortality risk (figure 2). However, without more studies of the TL–mortality risk association in ectothermic vertebrates using different methods it is impossible to dissect this suggestion further. The presence of an unexpected methodological difference in the association between TL and mortality risk highlights the need for more studies that apply both qPCR and TRF techniques to the same samples to understand how and why results might differ with methodology.

We found no evidence for effects of sex, age group or follow-up time on the association between TL and mortality risk. In humans and a handful of other mammals investigated to date, a general trend of longer TL in females than males has been observed and related to sex differences in lifespan commonly documented in polygynous species [61–63] (although see [64]). The vast majority of the studies included in our meta-analysis came from bird species, which tend to be monogamous and in which there remains limited evidence for sex differences in TL and lifespan [32]. To our knowledge, only one study to date has reported sex differences in the relationship between TL and lifetime reproductive fitness in any wild vertebrate and this study was in a polygynous reptile [33]. Although our analyses support the lack of a sex effect on the TL–mortality risk association in birds, further investigation of sex differences in systems exhibiting polygynous mating systems and sexual dimorphism is required before drawing any conclusions about the phylogenetic generality of this pattern. A previous meta-analysis found that the TL–mortality risk association declines with age within studies of healthy adult humans [9]. This result was interpreted as support for TL representing a better marker of the failure of somatic redundancy mechanisms rather than of biological ageing [9]. The fact that we found similar TL–mortality risk associations in both studies of juvenile and adults would support the idea that TL is not necessarily a biomarker of biological ageing. Furthermore, the lack of any association between TL–mortality risk association and species' maximum recorded lifespan suggests the observed association is not specific to particularly long- or short-lived bird species in our sample. Finally, the lack of any effect of follow-up period between TL measurement and assessment of mortality or recapture in our selected studies implies that TL predicts mortality just as well over short periods (e.g. to the subsequent year or breeding season) as it does over multiple years.

Our results provide support for the prediction that shorter TLs are associated with increased mortality risk in birds, but an important further question raised by this is what processes are responsible for this pattern. The association could be the result of individual differences, associated with genetic, epigenetic or developmental variation, which generate consistent differences in both TL and mortality risk across the lifetimes of individuals. In addition, cumulative experience of environmental stress or investment in growth and reproduction could simultaneously drive telomere shortening and increase mortality risk. The importance of among-individual differences in TL versus telomere shortening as predictors of mortality risk, while not mutually exclusive, remains a major question for researchers interested in telomere dynamics at the whole organism level. The human literature reveals TL to be moderately to highly heritable [21] and that self-reported experience of stressful events is associated with shorter TL [65]. One longitudinal study of different populations reported extremely high repeatability of TL within individuals across a period of a decade or so and argued that most of the variation in TL could therefore be attributed to genetic or early life factors [20]. The literature on non-model vertebrates, again very predominantly from birds, does offer evidence that rapid growth and physiological stress are associated with shorter TL [44,66]. However, estimates of the heritability of TL are very variable and often associated with very large confidence intervals, suggesting issues with power [67]. Furthermore, while some studies have identified telomere shortening as a predictor of survival [38,47], there is also evidence for associations between an individual's average TL and their lifespan [22,40,47]. Longitudinal studies capable of testing the degree to which survival and longevity are predicted by an individual's lifetime average TL or their rate of telomere attrition are now required to address this important question. Such studies can also help to establish whether TL measured in early life represents a better predictor of subsequent lifespan than later TL, as recently found in captive zebra finches (*Taeniopygia guttata*) [25]. In due course, the application of meta-analytic methods to the results of such longitudinal studies can provide consensus regarding when and how variation in TL predicts key components of organismal fitness.

## Supplementary Material

Supplementary Table 1

## Supplementary Material

Supplementary File 1

## Supplementary Material

Data

## Supplementary Material

Sex specific data

## Data accessibility

Data used in the meta-analyses are published as the electronic supplementary material.
